# Thinking Health-related Behaviors in a Climate Change Context: A Narrative Review

**DOI:** 10.1093/abm/kaac039

**Published:** 2022-07-21

**Authors:** Guillaume Chevance, Ujué Fresán, Eric Hekler, Donald Edmondson, Simon J Lloyd, Joan Ballester, Jill Litt, Ivana Cvijanovic, Vera Araújo-Soares, Paquito Bernard

**Affiliations:** ISGlobal, Barcelona, Spain; ISGlobal, Barcelona, Spain; Herbert Wertheim School of Public Health and Human Longevity Science, UC San Diego, San Diego, CA, USA; Center for Wireless and Population Health Systems, Qualcomm Institute, UC San Diego, San Diego, CA, USA; Center for Behavioral Cardiovascular Health, Columbia University Irving Medical Center, New York, NY, USA; ISGlobal, Barcelona, Spain; ISGlobal, Barcelona, Spain; ISGlobal, Barcelona, Spain; Environmental Studies, University of Colorado Boulder, CO, USA; ISGlobal, Barcelona, Spain; Health Technology & Services Research, University of Twente, The Netherlands; Department of Physical Activity Sciences, Université du Québec à Montréal, Montréal, Québec, Canada; Research Center, University Institute of Mental Health at Montreal, Montréal, Quebec, Canada

**Keywords:** Behavioral health, Lifestyle medicine, Sustainability, Global warming, Environmental changes, Planetary health

## Abstract

**Background:**

Human activities have changed the environment so profoundly over the past two centuries that human-induced climate change is now posing serious health-related threats to current and future generations. Rapid action from all scientific fields, including behavioral medicine, is needed to contribute to both mitigation of, and adaption to, climate change.

**Purpose:**

This article aims to identify potential bi-directional associations between climate change impacts and health-related behaviors, as well as a set of key actions for the behavioral medicine community.

**Methods:**

We synthesized the existing literature about (*i*) the impacts of rising temperatures, extreme weather events, air pollution, and rising sea level on individual behaviors (e.g., eating behaviors, physical activity, sleep, substance use, and preventive care) as well as the structural factors related to these behaviors (e.g., the food system); and (ii) the concurrent positive and negative roles that health-related behaviors can play in mitigation and adaptation to climate change.

**Results:**

Based on this literature review, we propose a first conceptual model of climate change and health-related behavior feedback loops. Key actions are proposed, with particular consideration for health equity implications of future behavioral interventions. Actions to bridge the fields of behavioral medicine and climate sciences are also discussed.

**Conclusions:**

We contend that climate change is among the most urgent issues facing all scientists and should become a central priority for the behavioral medicine community.

## Teaser

Based on a narrative review, this article proposes a first conceptual model linking climate change and health-related behavior

The purpose of this review is to address the field of behavioral medicine directly, and to communicate some of the risks, challenges, and “opportunities” that climate change poses to this field uniquely. In line with recent goals formulated by the Society of Behavioral Medicine, i.e., “*develop research questions at the intersection of climate change, behavior change, and health*” [1], the first section of this article presents basic information on climate change and how climate change is shaping our health-related behaviors. The present manuscript notably focuses on the problems caused by anthropogenic climate change, such as rising temperatures, increasing occurrence and intensity of extreme weather events, air pollution, and rising sea level. The second section illustrates how the promotion of sustainable health behaviors could aid climate change mitigation and adaptation. The last sections of this ­article focused on the crucial point of health equity and provide insight on how different types of equity should be addressed when thinking about the associations between health behaviors and climate change.

## Climate Change: Current Context and Impact on Health and Health Behaviors

Since the industrial revolution, between 1760 and 1820, human activities have changed our planet so profoundly that a new unofficial unit of geological time, called the Anthropocene, has been suggested to describe this era of Earth’s history, in which the effects of human activity are the dominant influence on climate and ecosystems [2]. We are now living in the seventh decade of the “great acceleration” that began post World War 2, characterized by pronounced, sometimes exponential, and arguably unsustainable growth in several indicators, such as human population, energy consumption, water use, transportation, and telecommunications. These human activities over the last two centuries have resulted in increased greenhouse gases (GHG) emissions and ultimately the sharp warming of our planet. Over the last decade, global surface temperature has increased by 1.09°C (0.95–1.20 °C estimated range), compared to the preindustrial period (1850–1900) [3]. An increase in global surface temperature above 1.5–2°C is considered extremely dangerous, due to the associated increases in the frequency of extreme weather events, massive biodiversity loss, serious disruptions to food and water safety, and multiple socio-economic consequences [4, 5]. Specific health risks include heat-related illnesses and mortality due to rising ambient temperatures; malnutrition or undernutrition due to reduced food quality and security; freshwater scarcity; together with other indirect effects such as increased violence due to resource scarcity; propagation of infectious diseases and vector-borne diseases; massive climate-induced population displacement; mental health risk (e.g., with more frequent exposure to extreme weather events); as well as illnesses caused by poor air quality [6–11].

Actions taken to date are insufficient for mitigating these threats. Stressing the urgency of the issue, the 1.5°C global temperature threshold is expected to be exceeded by 2040 under most scenarios of the Intergovernmental Panel for Climate Change (IPCC; [3]). Clearly, the next few years are the most decisive in human history [12]. Recognizing these threats, in 2015, the Lancet commission offered the Planetary Health framework to integrate the concept of planetary boundaries into our understanding of public health [6]. The Planetary Health framework proposes that human health and human civilization depend on flourishing natural systems and the wise stewardship of those natural systems and that overtaking planetary boundaries, particularly in relation to climate change and its impact, will exert increasingly harmful effects on human health globally.

## The Specific Impact of Climate Change on Health Behaviors

Although there are deep interconnections between each of the potential effects of climate change and associated health behaviors, it is typical in the literature to study each aspect separately, using a reductionist approach. In line with this, Table 1 summarizes the known relations between specific aspects of climate change and specific health-related behaviors. As a general view on these associations, we propose that climate change is associated with health behaviors through at least two different pathways: (i) direct and indirect (or mediated) effects and (ii) in the form of behavioral shocks and secular trends. Example of direct effects includes the impact of heat extremes on the human physiology and subsequent consequences in terms of sleep or physical activity, while indirect effects include the impact of hurricanes or typhoons on sleep quality via mental health issues (e.g., post-traumatic stress disorders) or on physical activity via the deterioration of sports infrastructures. Behavioral shocks and secular trends referred to the time scales at which climate change can impact behaviors, with post-extreme weather events situations of emergency (i.e., behavioral shocks), and slower secular changes in the climate impacting our behaviors on the long run such as the progressive temperatures increase and their behavioral effects. Of note, research on the specific impact of climate change on health behaviors is in its infancy because most previous research conducted so far have focused on outcomes such as mortality or hospitalizations. We believe that the behavioral medicine community has an important responsibility in accelerating the research about the impact of climate change on health behaviors, since a better understanding of these impacts should help to cope with.

**Table 1.** Summary of the Impacts of Climate Change on Health Behaviors.

	Eating behaviors and the food system	Physical activity and sedentary behaviors	Sleep	Substance use	Preventive care	Water consumption
Rising temperatures	Negative impact on crop yields and the nutritional quality of vegetables and legumes [13]; Could be dramatic in places where dietary diversity is low [14]; Increase food insecurity globally, since even the wealthiest nations often rely extensively on food imports [15]; Impact individual behaviors through food availability and prices [16]	Associations non-linear with tipping point (i.e., sharp decrease) around ~30°C, depending on other local factors, such as humidity [17–20]; Simulation studies suggest an overall negative impact on physical activity participation [21,22]; Particular threat for outdoor workers with productivity declining at 20°C [23,24]	Amplify sleep disturbances and obstructive sleep apnea, particularly in elderly, low-income populations and other vulnerable groups [25]; Nighttime temperatures registering above 25°C reduce individual sleep duration by over 7 min compared with 5–10°C [26]	Few studies available and mixed findings depending on the geographical context; Effects on alcohol potentially mediated by prolonged daylight [27–29]	Effects mediated by extreme weather events; see below	Effects mediated by rising sea level; see below
Extreme events	Harm agricultural production and threaten food supply-chain [30] and ultimately food prices [16]; Might be associated with unhealthy eating behaviors through mental health issues such as post-traumatic-stress-disorders [31]	Direct negative effect through the deterioration of sport and physical activity facilities as well as bike paths and pedestrian walkways [17, 32, 33]; Indirect negative effect through acute stress disorders [34]	Negative impact on sleep that can be mediated by mental health issues such as greater post-disaster states of fear or anxiety [25, 35–39]	Increase in alcohol use was observed after hurricanes [40–42]; We did not found significant associations with smoking [41, 43]; Effects moderated by history of traumatic experiences, gender and spirituality [44]	Negative impact on medication adherence, and preventive care via health systems breakdown [45,46]	Can disrupt and contaminate water supplies (e.g., via damaged drinking water wells), thus impacting fresh water accessibility and consumption [47]
Air pollution	Can accumulate in the food chain and potentially reduces worker productivity in the food sector [48]; Might also be associated to unhealthy food choices via neuro-biological mechanisms, such as neuro-inflammation and self-regulation [49]	Negatively associated with leisure physical activity and active transportation, and positively with sedentary behaviors, with pronounced effects among participants with respiratory conditions [17]; Negative impact of air pollution from wildfires on physical activity in children and adults [50–52]	Potentially associated with higher sleep disturbances such as snoring, sleep initiation and maintenance and sleep apnea [53–55]	Few studies available; Greater air pollution concentration was associated with a significant increase in emergency department visits for substance abuse during the following days [55] but this effect has not been replicated in a second study [56]	Potential association not mentioned in the present review	Potential association not mentioned in the present review
Rising sea level	Salt water intrusion into ground water supplies negatively impacts crops yields and food nutritional quality [57–59]; Daily sodium consumption of 5.2–16.4 g has been found in low-lying coastal countries, while the daily recommended dose is around 2 g [60,61]	Can negatively interact with extreme weather events and strengthen their direct negative effects on physical activity infrastructures	Living in a zone threatened by rising sea level can be associated with mental-health-mediated sleep issues [62]	Potential association not mentioned in the present review	Negative impact on preventive care (e.g., toilets or latrines use) has been observed because of forced-relocations [62]	Salt water intrusion threatens access to safe drinking water and increase individual’s sodium consumption

*Note*. A more detailed discussion of the issues in this article can be found in a preprint available at https://osf.io/pb8vc/.

## How Can Health Behaviors Influence Climate Change (for Better and Worse)?

As shown in the above section, climate change will increasingly shape our behaviors in the future. In return, our health behaviors could have a significant impact on climate change. This impact can be positive, by participating in activities targeting climate change mitigation and adaptation (e.g., [63]). It could also be negative, via engaging in health behaviors that increase a person’s carbon footprint (i.e., the amount of carbon dioxide released into the atmosphere as a result of the activities of a particular individual [64]).

## The Role of Health Behaviors for Mitigating Climate Change

Mitigation can be defined as proactive efforts to limit climate change [65]. Two specific health-related behaviors have notably been classified as potentially “high-impact” individual actions, because they have a major influence on GHG emissions: reduced meat consumption and active transportation ([66]; high-impact is in contrast to other behaviors such as sleep, which have not been linked as behaviors that could contribute to mitigating climate change).

Reducing meat consumption, notably ruminant meat (e.g., beef), is often mentioned as a high-impact proenvironmental behavior. Current meat production is responsible for substantial GHG emissions and requires significant land and freshwater use [67–69]. A recent systematic review estimated that shifting from a standard diet that includes meat consumption to an ovolactovegetarian diet (i.e., meat- and fish-free, but consumption of eggs and dairy products) or a vegan diet (i.e., total absence of animal-derived foods) would reduce individuals’ GHG emissions by an average of 35% and 49%, respectively [68]. A small amount of meat is compatible with sustainable diets (e.g., all together, one serving of red meat per week, 2 servings of white meat or fish, 1 serving of dairies per day, and 1.5 eggs per week), but in much lower quantities than that of the current trends [70]. From the perspective of what an individual could do, cutting down meat consumption is the most effective food system strategy for staying within planetary boundaries [71]. This mainly applies to high- and middle-income countries, in low-income countries an increase in animal product consumption may be needed to reduce malnutrition [72].

Active transportation, i.e., riding a bike or walking instead of driving an individual car, can contribute to the reduction of GHG emissions from the transportation system [73–75]. This is particularly critical since land transportation, and notably the use of individual cars, represents an important proportion of GHG emissions worldwide (e.g., 12% of total EU emissions [76] and 59% of the transportation sector emissions in the United States; the transportation sector accounts for 29% of all GHG emissions in the United States [77]). Estimates of GHG emissions reductions from interventions that promote active transport vary extensively from one study to another. For example, a quasi-experimental study conducted in New Zealand reported only a 1% reduction in CO_2_ emissions after three years of an intervention that combined walking and cycling infrastructure development and behavior change promotion at the scale of a city [78]. At a smaller scale, a case study conducted in Serbia showed that simple improvements to bicycle parking at a university reduced CO_2_ emissions associated with students’ transportation modes by 50% in one year, compared to the pre-intervention period ([79]; see [80] for a systematic review on this topic). There is notably a great opportunity for explicitly targeting short trips where motorized vehicles are not needed, as ~30% of car journeys in Europe cover distances of less than 3 km and 50% cover less than 5 km; these distances can be covered within 15–20 minutes by bicycle [81].

Of all the individual and health-related actions that one can initiate to mitigate climate change, reduction of meat consumption and active transport and are the most impactful for reducing individuals’ GHG emissions, based on the currently available evidence. This is true even in comparison to more widely publicized behaviors such as recycling or use of energy-saving appliances [63]. Studies of individual-level behaviors suggest that the most widespread “environmentally friendly” behaviors actually have low mitigation potential (e.g., recycling) and primarily serve to allow individuals to comfort themselves into believing that their current contributions are sufficient [65]. The scale and urgency of the climate change problem require that behaviors with a strong potential for carbon footprint reduction become the main focus of any interventions targeting a meaningful effect on the climate [82, 83]. Although this review does not argue for a particular way of implementing behavior change interventions, established frameworks in the field should be used to guide the development of behavior change interventions and identify mechanisms of co-beneficial behavior change (e.g., NIH Stage Model, MOST, ORBIT Model, SOBC experimental medicine approach, MRC Framework [84–87]). In comparison with traditional health behavior change interventions, a dual objective of health and sustainability should now be targeted in the field of behavioral medicine. In other words, future interventions should target human and planetary health simultaneously. This supposes further collaborations between experts in behavioral medicine and environmental scientists.

In regard of the potential effectiveness of individual (“bottom-up”) versus collective (“top-down”) actions to either mitigate climate change or change health behaviors, clearly, both are needed [88]. Individuals’ behavior change and large-scale/political actions need to be considered as interdependent and mutually influencing each other’s in a bi-directional way over time ([89]; and see [90] for the relevant concept of “spiral of sustainability”). High-impact individual behaviors should be enforced in political contexts that support decarbonation of the industrial system [90–92]. At the same time, pro-environmental behaviors can spread into, and ultimately shape, socio-ecological niches and social/cultural norms in a bottom-up fashion, thus leading to political and structural changes [93, 94].

## The Role of Health Behaviors for Adapting to Climate Change

The term “*adaptation*” corresponds to reactive responses that strengthen resilience and reduce vulnerability toward climate change consequences or, according to the IPCC, “the process of adjustment to actual or expected climate and its effects”. Previous studies have proposed that promoting particular health behaviors could help individuals and communities become more resilient and less vulnerable to future climate change consequences [66, 95].

Physical activity, notably, has been offered as an important factor for population resilience after certain types of extreme weather (see [17] for a review). For instance, Kirkpatrick et al [96]. described how bicycling enthusiasts have been organizing community events in US cities to demonstrate how bicycles may be useful just after flooding or hurricanes. Citizen bicyclists have developed community bike races named “disaster relief trials.” The riders use a cargo bicycle to haul large and fragile items, and must cross water, rough terrain, and physical barriers designed to simulate disaster conditions. These disaster relief trials are developed to improve community resilience and cohesion in case of extreme weather events. Other studies have reported physical education-based programs developed for children post-disaster. For example, a quasi-experimental trial was carried out in Leyte (Philippines), one year after the typhoon Haiyan, to test the effect of a school-based sport intervention on adolescents’ self-esteem [97]. The authors discussed the role of physical activity and sports for youth mental health post-typhoon (see also [98] for an example of physical activity programs post hurricanes in the United States). Physical activity and sport programs could also help social integration in the context of forced climatic migrations in the next years (see for an example [99]).

Beyond physical activity, adaptation with respect to eating behaviors has also begun in many places. For example, relatively successful examples of rooftop urban agriculture, providing fresh greens with shorter transportation routes and storage time for local markets, exist in urban settings (see for example in New York and Chicago [100] and resulting from the COVID-19 pandemic [101]). There is an evidence from Byzantium, World War II Britain, and the Soviet Union collapse that urban gardens increase resilience in times of crisis [102]. Studies have also shown positive effects of involvement in community gardens (defined as green spaces where individuals from more than one family grow food communally or side-by-side) on vegetable consumption, weight management, and well-being among vulnerable populations ([103, 104]; see also [105], for an ongoing quasi-experimental trial on the effect of community gardening on several health behaviors).

Finally, sleep health/hygiene should also be included as an integral part of any climate resilient system [25]. Sleep interventions could notably aid individuals in coping with the psychological side effects of extreme weather events, forced migrations within and between countries, and rising ambient temperatures [25]. In conclusion, and regardless of the context, behavioral medicine expertise can be key in fostering individual and community resilience. All these initiatives should be considered high-value behavioral levers to be promoted within a behavioral medicine field that adopts the planetary health definition (see [106]).

## The Potential, Unintentional, Amplification of Climate Change by Health Behaviors and the Field of Behavioral Medicine, and Strategies to Avoid It

Beyond these adaptation and mitigation effects, some health-related behaviors are also amplifying climate change, and all health behaviors are not environmentally sustainable behaviors. Some sports represent an important source of individual GHG emissions [17]. Aside from massive professional sporting events, most sport-related emissions are caused by motorized transport that is required to participate in the sport. For example, physical activity-related travel (e.g., driving or flying to practice a specific activity) represented 2.2%–26% of the annual carbon footprint of German active adults [64]. Furthermore, and beyond meat consumption only, individual eating behaviors represent a significant contributor to GHG emissions worldwide [14]. Eating behaviors have an impact on climate change from food production, storage, and packaging, to food processing, distribution, sales, and waste [14]. This impact is independent from a food’s nutrition quality, meaning that, in some cases, healthy food could contribute to climate change more than unhealthy food (e.g., [107]). Relatedly, tobacco consumption (in)directly amplifies pollution, as smokers release air pollutants (i.e., 6 trillion smoked cigarettes annually [108]). Furthermore, tobacco-related deforestation has occurred in low- and middle-income countries during the last few decades [109].

For these reasons, definitions of sustainable physical activity and sustainable diet have already been proposed in the literature and, in our opinion, should be adopted as default definitions of the behaviors to be promoted in the field of behavioral medicine (as the ideal to be reached whenever possible). Central to this shift is a recognition that individual health goals and planetary health goals are inherently intertwined. Bjørnarå et al. defined sustainable physical activity as “*activities that are conducted with sufficient duration, intensity and frequency for promoting health, yet without excessive expenditure of energy for food, transportation, training facilities or equipment. Sustainable physical activities have low environmental impact, and they are culturally and economically acceptable and accessible*” [110]. Similarly, a sustainable diet has been defined as “*diets with low environmental impacts which contribute to food and nutrition security and to healthy life for present and future generations. Sustainable diets are protective and respectful of biodiversity and ecosystems, culturally acceptable, accessible, economically fair and affordable; nutritionally adequate, safe and healthy; while optimizing natural and human resources*” [111]. Local, unprocessed, plant-based, and seasonal foods are usually more sustainable [112]. If the field of behavioral medicine does not actively utilize definitions of health behaviors that account for both the health of individuals and the planet, it is highly plausible that, as a field, we could unintentionally contribute to further exacerbating climate change.

Extending the potential unintended consequences of the behavioral medicine community’s default options, intervention modality choices could also unintentionally exacerbate climate change. Research has documented the carbon footprint of different intervention modalities, such as different modes of delivery for behavioral support for smoking cessation. The respective carbon footprint of text messages, telephone, group counseling, and individual counseling were 0.8, 0.9, 16.1, and 16.4 tons of CO_2_ equivalent for 1000 smokers, respectively [113]. As proposed elsewhere [114], we contend that researchers and clinicians should systematically consider both the short-term and long-term effects on health of individuals, and also the environmental implications of particular behavioral medicine research methods and interventions (see also [115] for strategies to reduce the environmental impact of clinical trials).

## Putting it All Together: A Model of the Feedback Loops Between Climate Change and Health Behaviors

One of our field’s dominant models for understanding and organizing the host of influences on health and health behavior is the socio-ecological model which encompasses individual, interpersonal, organizational, and public policy influences, as well as the natural environment [116]. It is meant to guide understanding of how human behavior interacts with other “levels” of influence. This paper argues that climate change is an all-encompassing influence on health and health behavior, as well as on each level of the socio-ecological model. We also argue that, with ongoing climate change, the impact of the natural environment on human health and health behaviors is likely to become increasingly important in the next years. Here, rather than merely highlight an additional “level” to the social-ecological model we propose an initial complex system model, in the form of feedback loops, to illustrate the reciprocal associations between health behaviors and factors related to climate change. While this model is only a rough starting point, we hope it can function as a bridge between the ways behavioral medicine has historically worked and how the field could transition to do its part in climate change mitigation and adaptation.

**Figure 1.**
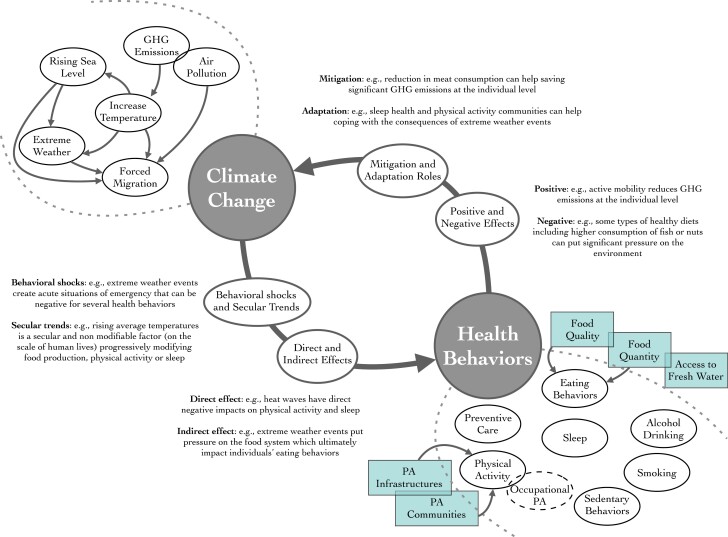
The Climate Change and Health Behaviors Feedback Loops Model. Note. PA = physical activity; the category “eating behaviors” refers here to the different behavioral sub-components identified in the literature review, such as the quantity and quality/type of food consumed by an individual; individual eating behaviors are influenced by structural factors related to food quantity and quality also named food production and security in the present review. GHG emissions and air pollution circles are intricated because GHGs are a type of air pollution.

Figure 1 synthesizes the associations previously described. This figure visualizes the feedback loops between climate change and health behaviors, highlighting that (i) climate change is shaping health behaviors (see Table 1) and that, in return, (ii) health behaviors might have different impacts on climate change outcomes. Based on the present article, we proposed that climate change is associated with health behaviors through at least two different pathways (lower-left corner of the figure): direct and indirect (or mediated) effects (e.g., the disruptive direct impact of heat waves on the physiology of sleep *versus* the indirect impact of extreme weather events on sleep through stress and anxiety) and in the form of behavioral shocks and secular trends (e.g., the short-term effect of an extreme weather event versus the long processes of temperatures increase; see Fig. 1). We propose that health behaviors are associated with climate change through two additional modalities (upper right corner of the figure): positive *versus* negative effects, as well as both mitigation and adaptation roles. Associations within climate change outcomes are mapped in the upper left corner of the figure. Individual health behaviors are represented by lines (except occupational physical activity, which represents a specific case materialized here in dotted line); structural factors influencing individual behaviors such as “physical activity infrastructure”, “food quality” and “access to fresh water” are framed outside of the “behavioral cloud” in squares. Although there is substantial evidence that health behaviors co-vary [117, 118], the between-behavior associations (lower right corner of the Figure) are not represented here for readability.

Relevant to the various distinctions made in Fig. 1, this article showed that the associations between climate change outcomes and health behaviors could be both uni- and bi-directional: (i) uni-directional associations include, for instance, the association between air pollution and sedentary behaviors (i.e., higher level of air pollution leading to a higher level of sedentary behaviors) or the negative association between extreme weather events and sleep (i.e., extreme weather events have an impact on sleep, not the other way around); (ii) notable bi-directional associations (i.e., self-reinforcing feedback loops) exist between active transportation and air pollution (i.e., higher level of active transportation is associated with less local air pollution, which in turn might increase active transportation), or between GHG emissions and food quantity and quality (i.e., growing food requires the production of GHG, which negatively impacts food quality). These specific associations are not mapped in the present Figure but could be useful when addressing the complexity between climate change and health behaviors.

## Health Equity: A Crucial Aspect of Future Behavioral Medicine Interventions Targeting Climate Co-benefits

Climate change raises health equity issues that are crucial to consider when thinking about the associations between climate change and health behaviors. Within countries, people who are wealthy produce more GHG emissions, while people who are socially disadvantaged emit less GHG but have fewer resources to cope with present and future climate change consequences. Indeed, annual incomes are a major determinant of household GHG emissions [119, 120]. For example, US households with more than $100,000 annual income are responsible for nearly one-third of all households’ total carbon footprint in the country, but account for only 22% of the US population [121]. In parallel, it is expected that people living in socially disadvantaged areas, or with less financial resources, will experience more difficulties coping with the health consequences of climate change [122]. For example, a study conducted in San Francisco showed that residents with low income, including no private motor transport, used more nonmotorized transportation during extreme heat episodes, potentially exposing them to a higher risk of heat-related health issues than residents with higher incomes [123]. Furthermore, there is an evidence that individuals living in socially-disadvantaged areas are more exposed to environmental pollutants (e.g., air, water, land, and noise; which are negatively associated with health behaviors; see for example [124]). The field of behavioral medicine has the knowledge, skills, and capacities to meaningfully contribute to reducing health disparities. A key next step for the field could be to consciously develop interventions that are culturally, contextually, and economically appropriate for historically underserved communities and populations, and that support these communities in adapting to climate change. In contrast, mitigation interventions should primarily target individuals with high incomes, as they are proportionally contributing more to GHG levels.

Between countries, a similar pattern is evident, as low- and middle-income countries are more likely to suffer the adverse consequences of climate change than higher-income countries in the short term [125]. This pattern raises health equity issues at a global scale since the major direct contributors to global warming are mostly from higher-income countries [126]. The top 10% of high-income countries cause 33% of global GHG emissions, whereas the bottom 50% are responsible for only 15% of global emissions [127]. Today, excluding some very specific oil-producing countries (e.g., Qatar), wealthy Western nations, such as Australia, the United States, and Canada have the highest per capita footprint, between 15 and 17 tones of CO_2_ per person and year. To achieve the goal of the Paris agreement, each individual’s carbon footprint must stay below 2 tones of CO_2_ per year [91]. Per capita emissions cannot be solely explained by fossil fuel infrastructure (e.g., in 2019, 3.6% of France’s energy came from fossil fuels, compared to 20% in Germany [128]). These estimates seem to demonstrate that Western societies have an ethical and moral responsibility to shift their practices, given their historical and present-day contributions to the problem. It is clear that most of the behavioral changes necessary to mitigate climate change must be made by high-income countries. In parallel, high-income countries must increase their support to low- and middle-income countries, to aid their efforts to cope with the current and future consequences of climate change (see [90]).

Health equity issues also arise at the inter-generational level. Major negative consequences resulting from today’s GHG emissions will be experienced by today’s children and young people who currently have little control over those emissions (i.e., 40–50 years from now), not today’s adults [129]. Indeed, there is a temporal delay between most GHG emissions (i.e., notably CO_2_) and ecosystem degradation [3]. In other words, the future health of a child born today, and that of their children, is harmed by our current GHG emissions [130]. A recent study suggests that children born in 2020 will experience a two- to sevenfold increase in extreme events, particularly heat waves, compared with people born in 1960 [131]. If we do not radically limit our emissions, many future generations will be asked to cope with unprecedented challenges. The most consequential decisions today are almost certainly being influenced by intergenerational delay discounting (i.e., countries’ focus on short-term gains vs long-term gains). Moreover, high-income countries’ current approach to emissions reductions since the Paris Agreement transfers a significant proportion of the mitigation burden to future generations. Future generations will face the challenge of coping with increasing climate change-related health impacts, while simultaneously developing a low/no carbon energy system, with fewer resources to do so and in a potentially more unstable (i.e., at the social and ecological levels) world [132]. The field of behavioral medicine, with its knowledge of the psychology of these and other issues, could play a major role in helping to advance intergenerational equity as a guiding principle for motivating more rapid efforts on climate change mitigation and adaptation explicitly designed to support children and future generations [133].

Finally, climate change is expected to impact the health of women and men differently, particularly in low- and middle-income countries (see [134]). For example, rising temperatures could substantially worsen the health impacts of menopause, notably hot flashes [135]. Furthermore, climate change has the potential to impact women’s health through perturbation in the timing of menarche (i.e., the first occurrence of a woman’s menstruation) which, in turn, affects women’s risk of diseases (see [136] for the pathways including impact through food, stress or exposure to environmental pollutions). Men could experience risk of some impacts like suicide and severe depression due to extreme weather events impacting their occupational activities (e.g., droughts in Indian farmers [137]) differently than women. Based on this, the field of behavioral medicine should consciously monitor and seek to understand and address any disparities that might manifest in relation to sex or gender.

## Bridging Behavioral Medicine and Climate Sciences

It is urgent for behavioral medicine to actively seek synergies between behavioral medicine and climate research communities. Several fundamental principles of behavioral medicine should facilitate this movement (see [138, 139]), for a conceptual definition of behavioral medicine). Indeed, behavioral medicine (i) is a highly interdisciplinary field that acknowledges multiple influences related to health behaviors (i.e., assumes individuals are embedded within their larger social and natural contexts); (ii) uses reciprocal determinism to characterize the relationships between contexts and behaviors (i.e., understands that both shape each other); and (iii) is based on seeking fundamental principles about human behaviors, but with a recognition that said principles influence and are influenced by cultural and environmental factors. We thus contend that behavioral medicine, as a scientific discipline and community, would benefit from opening more lines of research concerned with planetary health, notably in the context of climate change.

As a first step toward merging traditional behavioral medicine and planetary health approaches, we recommend that the definition of health behavior be revised to include the notions of carbon, water, and ecological footprints as determinants and consequences of health and health behavior broadly, as has already been proposed for physical activity and eating behaviors. We contend that the concept of health equity should also be added to this definition. To support merging the fields, we propose an update of Gochman’s definition [140]: “*health-related behaviors can be defined as actions and patterns of actions within a context that enable human choices that result in reduced or net-zero carbon, energy, water, and ecological footprint and (in)directly result in equitable improvement, restoration, and maintenance of health for every humans and other living beings’ health for current and future generations*”. As mentioned before, if the field of behavioral medicine does not actively utilize definitions of health behaviors that account for both the health of individuals and the planet, it is highly plausible that we could unintentionally contribute to further exacerbating climate change.

## Conclusion

More empirically supported actions are needed from the behavioral medicine community to (i) better cope with the impacts of climate change on health behaviors and (ii) foster the role of sustainable health behaviors in communities’ efforts toward climate change mitigation and adaptation. It is clear that climate change is a risk multiplier of current and future unhealthy behaviors. The current major risk for behavioral medicine is to do “too little, too late” to address climate change. This review has shown that all behaviors which accelerate climate change should be considered unhealthy behaviors. The behavioral medicine community must transform so that our expertise can help future generations live well on a finite planet [141, 142].
